# Preventive Intervention to Prevent Delirium in Patients Hospitalized in Intensive Care Unit 

**Published:** 2018-04

**Authors:** Padideh Ghaeli, Fatemeh Shahhatami, Mojtaba Mojtahed Zade, Mostafa Mohammadi, Mohammad Arbabi

**Affiliations:** 1 Department of Clinical Pharmacy, School of Pharmacy, Tehran University of Medical Sciences, Tehran, Iran.; 2 School of Pharmacy, Tehran University of Medical Sciences, Teheran, Iran.; 3 Director of Intensive Care Section, Imam Khomeini Hospital, Tehran University of Medical Sciences, Tehran, Iran.; 4 Director of Neuropsychiatry Section of Iranian Psychiatry Association, Roozbeh Hospital, Tehran University of Medical Sciences, Tehran, Iran.

**Keywords:** *Complication*, *Delirium*, *Intensive Care Unit*, *Non-Pharmacological*, *Prevention*

## Abstract

**Objective:** Delirium is a clinical syndrome associated with multiple short- and long-term complications; therefore, prevention is an essential part of its management. This study was conducted to review the effective non-pharmacological interventions that can reduce the incidence or duration of delirium in critically ill patients.

**Method** **:** A search was made in PubMed, Scopus, Psych INFO and Google Scholar databases without any time constraints. The information available was collected and sorted, and a secondary study of narrative review was done. The views of specialists on this topic were received via email and included in the texts and recommendations.

**Discussion:** Delirium is a common, costly and potentially damaging illness in patients who are staying in hospitals, especially older patients in ICU. Thus, preventing delirium could be one of the most effective methods in preventing the complications. The present study aimed at conducting a review-validity study to generate a general view on the activities which might be effective in preventing delirium in patients.

Delirium is a disorder characterized by acute brain dysfunction that occurs frequently in patients who are admitted to acute care hospitals (1). Delirium is defined in Diagnostic and Statistical Manual of Mental Disorders, 5^th^ edition (DSM-5) (2) as a global disturbance of consciousness characterized by fluctuating mental status, inattention, and disorganized thinking, which develops over a short period of time and tends to fluctuate throughout the day. Critically ill patients have an increased risk of developing delirium during their hospital stay. This often results from sepsis and disturbances in inflammation and coagulation pathways leading to microvascular thrombosis (3).

In addition, critical illness disrupts circadian rhythm and sleep patterns; moreover, along with sedatives such as benzodiazepines that are commonly used to treat delirium in septic patients, can impair immunity and contribute to delirium (4, 5).

The overall incidence of delirium in patients in critical care is reported to be about 30%, but it is 60% to-80% in sedated ventilated patients, excluding those admitted after major elective surgery (6-8). A point prevalence study of 497 patients in ICU in 11 countries showed that 68% of patients were either over sedated or had delirium (9). 

The high occurrence rate of delirium among hospitalized patients and its consequences have made it a serious health concern.

Among patients requiring admission to an ICU, delirium is associated with an increased length of hospital stay, increased costs and complication rates (6, 10-14). Although causality between delirium and mortality is not established, critically ill patients who develop delirium are up to 3 times more likely to die within 6 months than those who do not (6), with each additional day of delirium being associated with a 10% increase in the risk of death (11). Importantly, in mechanically ventilated patients, delirium might be associated with long-term cognitive impairment (15). 

To date, there has been no evidence to support this idea that pharmacological interventions can prevent delirium. However, several non-pharmacological intervention studies have been shown to be effective. Hence, preventing delirium is the most effective strategy to reduce its frequency and complications (16). In this article, a review of some of the evidence will be sought from published data in relation to non-pharmacological interventions.

## Materials and Methods

This search was made in PubMed, Scopus, Psych INFO, and Google Scholar databases without any time constraints. The information available was collected and sorted, and a secondary study of narrative review was conducted. The views of specialists on this topic were received via email and were included in the texts and recommendations. The recommendations were scored as follows: systematic review of randomized clinical trials (RCT8), IA score, the RCT study (IB), systematic review of cohort (2B), cohort studies (2B), systematic review of case control (3A), case control (3B), systematic review of case series (4A), the case series or cross section studies (4B) and other studies (5).

***Preventive Measures***

Preventing delirium means using methods that can effectively decrease the risk of delirium incidents and ultimately, cause improvement in clinical outcomes in geriatric patients who show risk factors that may serve as the basis for delirium manifestation. To date, few clinical studies have been published on preventing delirium; nevertheless, they have already indicated that around 30% to -40% of delirium episodes are preventable (17-19).

Paying attention to risk factors that make grounds for delirium manifestation in individuals exposed to increasing risk of delirium (such as old patients) could prevent delirium incidence and could ultimately improve the clinical outcomes of these patients (19-21). Immobility, using physical constraints, using bowl catheter, malnutrition, psychedelics, some types of drugs, associated diseases, and dehydration in the individual can cause delirium symptoms (22). Old age, severe illness, dementia, physical frailty, infection and/or dehydration, vision impairments, drug interference caused by polypharmacy, surgery, and excessive use of alcohol are among other risk factors for delirium (23-26). The core of studying the non-pharmacological interventions in preventing delirium is based on identification, followed by managing the precipitating factors and/or delirium risk factors.

One of the most well-known studies on preventing delirium is the study of Inouye et al, also known as Yale Trial (18). In fact, Yale Clinical Trial was the first controlled clinical trial that showed there are other non-pharmacological ways to prevent delirium in geriatric patients. This intervention included employing a standardized protocol on taking medical measures to eliminate or reduce the 6 risk factors of delirium in individuals older than 70 years. The 6 delirium risk factors in this study were cognitive impairment, sleep deprivation, immobility, visual impairments, hearing impairment and dehydration. The results of this study showed that delirium symptoms were 9.9% in intervention group in comparison with the 15% in usual-care group. The total number of delirium and the total number of its episodes showed a significant decrease in the intervention group. Nevertheless, the intensity of delirium and its recurrence did not show any significant difference. This intervention was associated with considerable improvement in the degree of cognitive impairment manifested in patients with cognitive impairment at admission as well as significant reduction in the rate of use of sleep medications in all patients in the intervention group. Few years later, the study was modified and its defects were removed that changed it into a care model named HELP (Hospital Elder Life program), which is now practiced in hospitals by aiming at prevention of cognitive and functional disorders and defects in hospitalized geriatric patients (27, 28).

Mark Antonio et al in a similar study showed the effects of counseling services for geriatric patients in decreasing delirium syndromes in patients diagnosed with hip fracture (19). In their study was a prospective, randomized, and blinded study, they evaluated 126 patients aged 65 years and older who had a history of emergency hip surgical operations. The counseling targeted the 10 biggest risk factors in delirium in the geriatric patients including oxygen delivery to central nervous system (CNS), fluid and electrolyte balance, treatment severe pain, elimination of unnecessary drugs, regulating bladder and bowl function, adequate nutritional intake, early mobilization and rehabilitation, appropriate environmental stimuli, treatment agitated delirium, prevention, early detection, and treatment of postoperative complications. The results of their study revealed that the prevalence of delirium significantly decreased in the intervention group during hospitalization. The amount of delirium symptoms in a group who received adults counseling was 32% and in the control group who received the usual care was recorded to be 50%. In the intervention group, the risk of delirium and intensity of delirium decreased significantly. Therefore, a multi-purpose intervention can help reduce delirium in geriatric patients, improve quality of their care and treatment, and reduce their dysfunction. The advantage of this plan is that it can be easily implemented without increasing the health associated costs (29, 30). 

The effective role of early physical activity and rehabilitation in decreasing delirium risk, as claimed by both studies has been proved in both studies. Due to the importance of this factor, primary stimulation was performed as the first study on the non-drug prevention of delirium in the ICU patients to improve their performance. There were 2 other studies on this area that assessed the efficacy of combination of daily interruption of sedation with physical and occupational therapy and its effects in functional outcomes in patients receiving mechanical ventilation (31, 32). After the 2 studies were conducted, the researchers found that considerable reduction in delirium incidence, deep sedation, and reduction in the staying time in the intentive care unit and hospital were associated with increase in the number of ventilation-free days. Therefore, to decrease the prevalence and duration of delirium, it is recommended that geriatric patients in ICU perform primary mobility in the first possible opportunity.

Controlling environmental factors is one of the effective ways in delirium control and improvement. Although there is no empirical evidence to show that environment by itself can cause delirium, it seems specific environments might intensify delirium. In general, environmental factors play a significant role in creating risk factors in delirium development; therefore, if those factors can be managed by some approaches, it will be possible to decrease the risk of delirium symptoms (33-36).

Noise is one of the disturbing environmental factors. There are many noises in hospitals, especially in ICU, noises are caused by apparatuses, pumps, ventilators, alarms and/or the sounds of doing resuscitation. The noise in ICU has been recognized as a factor that disturbs sleep and disturbance in natural sleep, cycle might increase delirium symptoms (37). To prove this claim, the effects of noise on quality of sleep and delirium development was studied in a clinical trial in which headphones were used to protect the patients against hearing noise during night sleep. The results of the trial showed that the practice improved sleep and reduced confusion in patients (4). In addition to interference with patients’ sleep and rest, the noise might stimulate and intensify the symptoms for the delirium patients who are suffering from confusion and dizziness. Thus, efforts must be made to reduce those noises as much as possible. Furthermore, in patients who are in verge of seizure and might suffer from seizure attacks with the least number of stimuli including hearing stimulation, it is better to protect their ears by headphones so they cannot hear the noises around them. In another word, silence is the best sound for these patients. On the other hand, low environmental stimuli can leave the patient with his/her delirium state. Therefore, a completely calm atmosphere can cause delirium intensification and shall not be created unless in specific cases which have already been described (38). 

In addition to efforts to eliminate disturbing noises in the environment, making the place soothing and peaceful by playing relaxing music, could help reduce confusion and delirium in patients, especially for mechanical ventilation treatment patients who receive sedatives and are provided with conditions to tolerate the system and stay relaxed. Because they are still attached to the surrounding environment through hearing, using soft music with no lyrics could be helpful in lowering their anxiety and confusion caused by their physical conditions (39-41).

Using pleasant scents and air fresheners can be helpful in making the environment desirable for the patient. Although no study with high statistical population and suitable planning has been published on this topic, it seems that efforts to reduce disturbing environmental smells such as detergents and disinfectors, which are common in hospitals and replacing them with scents and air fresheners, could be helpful in preventing delirium and reducing confusion in patients (38, 42). 

Being placed in an environment where the changes in day and night hours are not noticeable contributes to sleep disorder and loss of alertness cycle, leading to, intensification of fatigue and confusion in patients. To improve these conditions, there are windows built in some ICUs to enable the patients to see changes in day lights and nights. In addition, in a room which might be dark at night, a dim light can reduce patient’s confusion at night. To help the patient on better perception of the 24-hour cycle, the patient should be provided with an analogue clock that shows the 24 hours of day and night and a calendar visible by the patient (38, 42). 

Therefore, it is important to provide the patient with a fair amount of visual, hearing and speech mobility to save him/her from disconnection from the surrounding environment, as, this disconnection can cause confusion and delirium in the patient. Furthermore, delirium is intensified by sensory disorders including vision impairments (24) and hearing impairment (43). For this reason, returning patient’s glasses and/or hearing aid might help prevent delirium.

Unfortunately, although the value of environmental interventions has been recognized and proven to some extent, they are not practiced enough (44).

Along with environmental elements, the emotional reaction of the patient to delirium symptoms and understanding the cognitive impairments could also serve as intensive factors in delirium. The patients must be told that their delirium symptoms are temporary and reversible and they do not develop permanent psychological disorder. The patients should be informed that delirium symptoms are associated with a specific illness, surgery, or other treatments and those symptoms are common and reversible; of course, except in conditions when delirium has been caused by brain stroke or major damages to the brain or other reasons which caused permanent brain damage to the individual and the patient is no longer able to understand and perceive words and sentences. Teaching patient’s friends and family about delirium is very useful as they might get concerned about delirium and its signs. 

In addition, educational programs have been used for work therapy and care staff has been used to prevent delirium solely or as a part of a multi-purpose intervention (45-50). There is evidence that an educational plan as an independent intervention can lower delirium symptoms (43, 49). Providing basic information on delirium prevalence and outcomes, teaching methods of evaluating delirium, introducing guidelines on treatment measures of the diseases through workshops and educational rounds could have significant role in preventing delirium are of paramount importance (47). Nevertheless, the format, time, duration and contents of topics that must be presented, as well as the special target group on which intervention should be done have not yet been specified fairly; however, in general, an education program for hospital staff and nurses that focuses on the evaluation prevention and treatment of delirium could reduce delirium symptoms and subsequently reduce the number of hospitalization days.

## Recommendations

To prevent delirium symptoms in ICU patients the followings are recommended:

1. The patient should be provided with the opportunity of receiving physical therapy, occupational therapy, and performing primary mobility in the first possible chance (IB).
2. Disturbing noises in the patient’s environment should be decreased too improve patient’s sleep and rest (IB).
3. Soft and soothing music should be played for the patient to make the environment pleasant and to reduce anxiety and confusion caused by his/her physical conditions (IB).
4. Pleasant fragrance and scents should be used to make the environment pleasant for the patient (5).
5. A clock should be placed in view of the patient along with a calendar to help him/her with lower confusion and improve communication with the surrounding environment. In addition, if possible, the patient’s bed should be placed close to the window so he/she can notice the change in the morning and night light, and if not, changes should be made in lighting based on the morning and night light (IB).
6. Glasses for patients with vision impairments and hearing aid in patients with hearing impairment will help them communicate better with the environment and reduce their confusion (IB).
7. Arranging for educational classes for work therapy on delirium recognition, prevention, and diagnosis is also very helpful (IB).

## Conclusion

Delirium is a common, costly and potentially damaging. Thus, preventing delirium could be one of the most effective methods in preventing the complication. To help prevent delirium, try to minimize modifiable risk factors and precipitating factors that is discussed in detail in this article.
